# Acute Cardiogenic Shock Requiring Extracorporeal Membrane Oxygenation Secondary to Catecholamine-Secreting Paraganglioma: A Case Report

**DOI:** 10.7759/cureus.79696

**Published:** 2025-02-26

**Authors:** Mohammed Salameh, Katelyn Swizer, Abhishek P Patel, Matei Petrescu

**Affiliations:** 1 Pediatric Critical Care Medicine, University of Texas Southwestern Medical Center, Dallas, USA; 2 Pediatric Hematology and Oncology, University of Michigan, Ann Arbor, USA; 3 Pediatric Critical Care Medicine, Baylor College of Medicine, San Antonio, USA

**Keywords:** cardiogenic shock, ecmo cpr, extra-adrenal paraganglioma, paraganglioma, pediatric shock, va-ecmo

## Abstract

A previously healthy 16-year-old female presented with acute onset of signs and symptoms of headache, nausea, sweating, abdominal pain, palpitations, chest pain, hypertension, and tachycardia. The patient was admitted to the pediatric critical care unit and rapidly progressed to cardiorespiratory failure, necessitating veno-arterial extracorporeal membrane oxygenation (VA ECMO) as a life-saving measure. After three days of ECMO support, complicated by arterial thrombi to the left lower limb, necessitating below-knee amputation, the patient was weaned off ECMO and ventilator support. A renal ultrasound was performed, as the patient had significant hypertension, which revealed a left para-renal mass. A biopsy of the mass, elevated plasma/urine catecholamine, and genetic testing confirmed the diagnosis of paraganglioma-pheochromocytoma (PG-PH) syndrome. A diagnosis of PG/PH was made, and a catecholamine surge due to the tumor was deemed the cause of the cardiac arrest. This is a rare disease, and a presentation with cardiorespiratory arrest has not been reported in children prior to this case. Our case highlights the importance of early identification of rare cases using history and examination, along with screening of those at high risk. It also shines a light on the importance of VA ECMO support in severe cases that present with cardiac arrest.

## Introduction

Pheochromocytomas (PHs) and paragangliomas (PGs) are catecholamine-secreting tumors, presenting with hypertension, palpitations, and diaphoresis, incidentally or on screening [1-4]. Multi-organ failure is a potentially lethal complication, with hypertension, circulatory shock, and involvement of the cardiopulmonary, neurologic, and reno-hepatic systems [5]. Pheochromocytoma crisis (PC) can occur spontaneously or by manipulation of the tumor, medications, trauma, or stress [6]. PC has high mortality, primarily cardiovascular, despite aggressive therapy. Refractory cardiogenic shock with undiagnosed PC is a complex situation to manage, with a broad differential. We report a 16-year-old female who presents acutely in cardiogenic shock, requiring veno-arterial extracorporeal membrane oxygenation (VA ECMO) as a life-saving measure. It highlights maintaining a diverse differential in a critically ill child, and essential considerations when initiating ECMO as a bridge to resection in a population with sparse data in the literature.

This article was previously presented as an oral presentation on September 30, 2021, at the ELSO Virtual Meeting.

## Case presentation

A 16-year-old female was transferred with acute chest pain and less than one day of headache, nausea, emesis, and weakness. She developed shortness of breath, diaphoresis, and palpitations. An electrocardiogram (EKG) obtained at the pediatrician’s office showed sinus tachycardia without ST changes. She arrived at the emergency room tachycardic, hypertensive, and increasingly hypoxic. On examination, her heart rate ranged from 130-150, blood pressure was 137/70, and oxygen saturation was 92-94% on a non-rebreather mask. On telemetry, she was thought to have supraventricular tachycardia (SVT), which did not respond to adenosine or esmolol. The EKG findings were suggestive of sinus tachycardia, with intermittent evidence of a right bundle branch block (RBBB) pattern and no change in rate. A chest computed tomography (CT) scan was negative for embolism, showing bilateral lower lobe ground-glass opacities. She was intubated due to impending respiratory failure and became increasingly hypotensive. The primary diagnosis was shock, secondary to possible myocarditis (Table 1).

**Table 1:** Troponin levels and trends on presentation and during admission

Day	Result	Units	Reference
32	0.25 H	ng/mL	0-0.03
21	6.28 H	ng/mL	0-0.03
11	4.01 H	ng/mL	0-0.03
8	0.28 H	ng/mL	0-0.03
4	6.03 H	ng/mL	0-0.03
3	12.79 H	ng/mL	0-0.03
1	17.09 H	ng/mL	0-0.03

In the pediatric critical care unit, she was febrile, with metabolic acidosis, transaminitis, acute kidney injury (AKI), and arrhythmia. Mean arterial pressure downtrended to the mid-30s, requiring inotropic support with epinephrine and norepinephrine at a maximum dose of 0.2 mcg/k/minute. She developed ventricular tachycardia, requiring defibrillation and four rounds of cardiopulmonary resuscitation (CPR) with return of spontaneous circulation (ROSC). Echocardiography showed severely depressed bi-ventricular function. She had decompensated heart failure, with acidosis and abnormal renal and hepatic functions.

**Figure 1:**
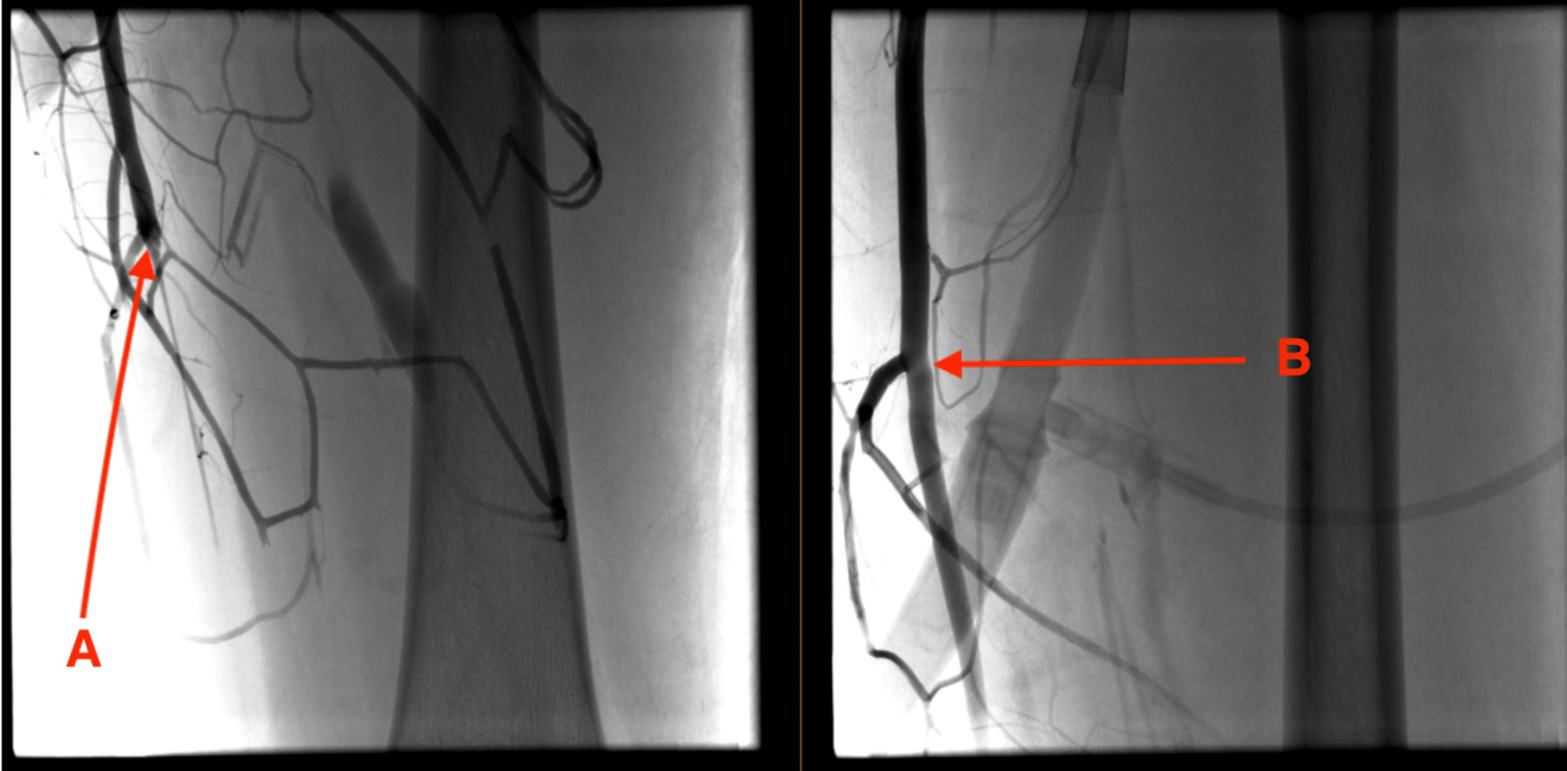
Angiography of the left femoral artery Arrow (A) shows narrowing of the left femoral artery; Arrow (B) shows recanalization of the left femoral artery.

VA ECMO was promptly initiated due to cardiovascular instability. Due to her acute decompensation, she was cannulated percutaneously using a femoral approach. However, percutaneous distal arterial cannulation of the left lower extremity was unsuccessful, and she required a cut-down left distal femoral cannulation to aid in perfusion. After the initiation of ECMO, the left leg was increasingly pale, and there was concern for compartment syndrome. Following bedside flow restoration, angiography revealed interruption below the knee (Figure 1). The femoral cannula was removed, and a similar-sized, suitable common carotid arterial cannula was placed.

Additionally, given the suboptimal opening of the aortic valve on echocardiography and left atrial dilation, she underwent atrial septostomy for left atrial decompression to help maintain existing heart function. Vascular surgery removed extensive clots from the left popliteal and femoral arteries and placed a new distal perfusion cannula. Orthopedic surgery performed a fasciotomy of the left thigh/lower extremity. While on ECMO, the patient was weaned off epinephrine as tolerated, to allow for decreased myocardial oxygen consumption and facilitate recovery of cardiac function. She remained intubated and with continuous renal replacement to optimize fluid balance and reduce cardiac work during recovery. Serial echocardiograms demonstrated a slow return of left ventricular ejection fraction, and she was weaned off vasopressors and decannulated after an 80-hour run on VA ECMO. The patient required a left above-the-knee amputation as a complication of compromised perfusion to the left lower extremity, presumably from shock presentation complicated by arterial thrombosis and prolonged impaired left lower leg perfusion.

Post-ECMO, milrinone and carvedilol were started to restore heart function, facilitate afterload reduction, and promote cardiac remodeling. Epinephrine and labetalol were used throughout her course to assist in heart recovery. Frequent echocardiograms were performed to assess the recovery of heart function.

**Figure 2:**
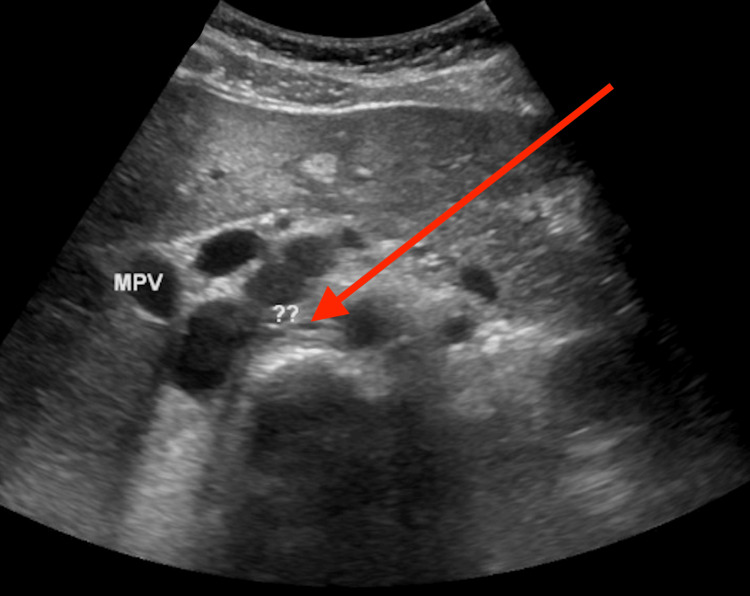
Suprarenal mass on ultrasonography The arrow refers to the suprarenal mass.

**Figure 3:**
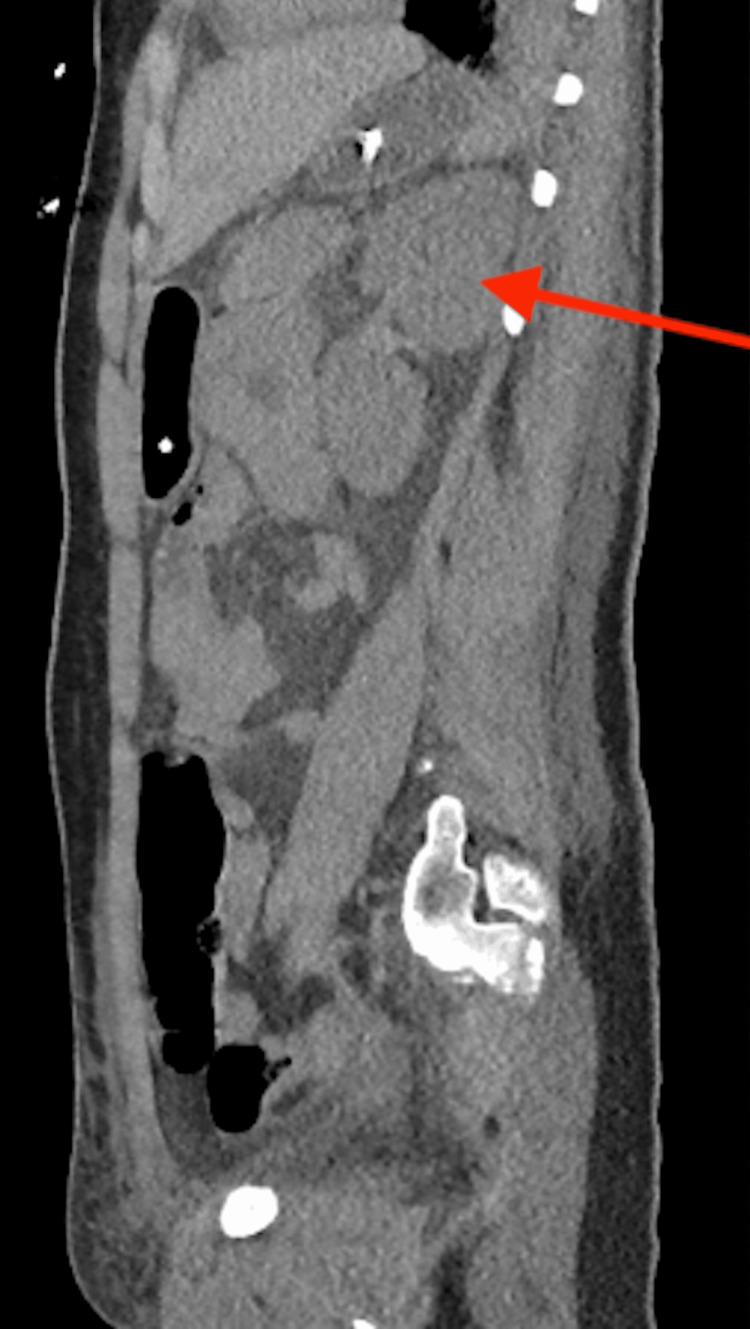
Left suprarenal mass on CT abdomen and pelvis The arrow shows the left suprarenal mass. CT, computed tomography

**Figure 4:**
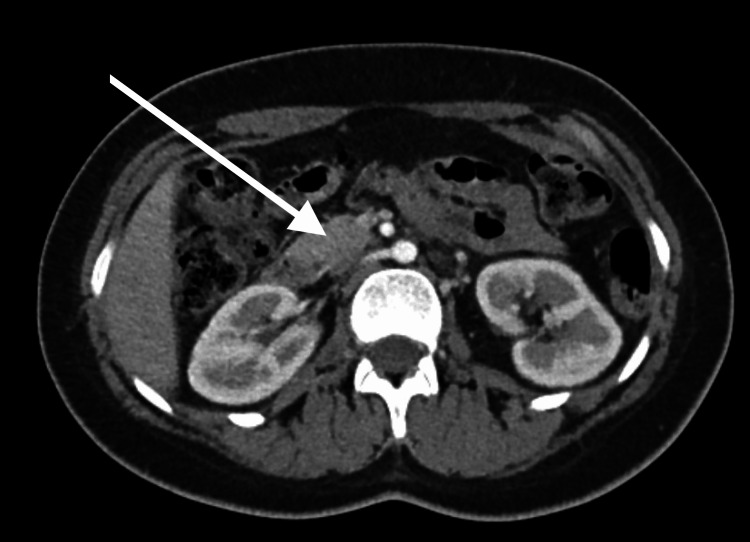
Tumor anterior to right kidney The arrow shows a tumor that is located anterior to the right kidney.

**Figure 5:**
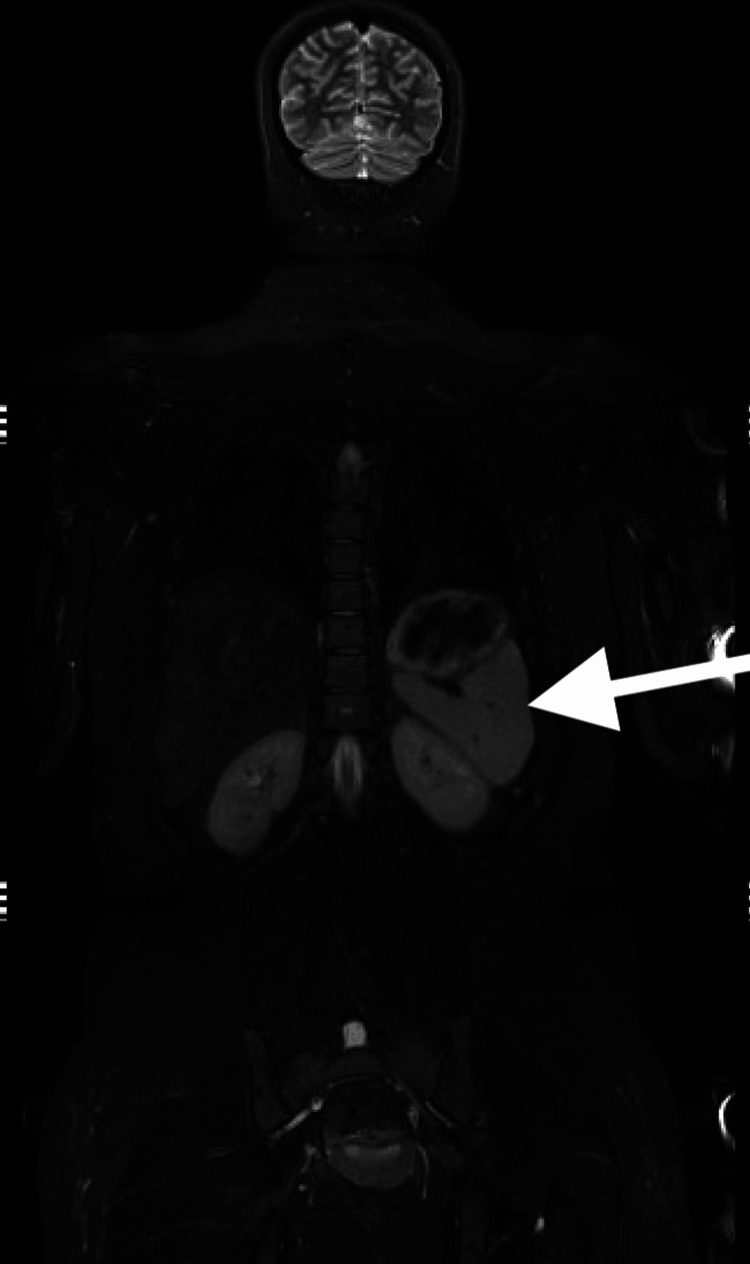
Left suprarenal mass The arrow refers to a mass infero-posterior to the left kidney.

She had persistently elevated blood pressure off ECMO, requiring multiple medications, including esmolol and nicardipine drips, with no response to maximum doses. Renal ultrasound and abdominal CT scan were done secondary to persistent AKI and hypertension, showing a 3.4 cm posterior pancreatic mass and a 4.8 x 5.5 cm inferior left kidney mass (Figures 2-3). Elevated urine vanillylmandelic acid and normetanephrine supported a diagnosis of PH/PG. Magnetic resonance imaging (MRI) without contrast was obtained and showed a solid tumor anterior to the right kidney (Figure 4) and a tumor infero-posterior to the left kidney; necrosis was also shown (Figure 5).

The patient was extubated nine days after decannulation. She continued to have significant hypertension, requiring multiple anti-hypertensives, including alpha and beta-blockers. She tolerated resection of both masses, with a period of normotension without antihypertensives and subsequent mild hypertension. At the time of discharge, her urine metanephrines were normal, and she was subsequently weaned off antihypertensives three weeks after surgery.

## Discussion

The classic triad of PH/PG is headache, palpitations, and sweating; however, many patients present atypically [1-4]. PC is another common presentation in medical literature [7]. Most present with several weeks or more of symptoms, or multiple documented episodes of hypertension before PC [7]. Our patient presented acutely, with no prior trauma, medical history, or medication use. Approximately 20% of neuroendocrine tumors are familial, with genetic syndromes more often found in children [4]. Whole exome sequencing showed that the patient had a succinate dehydrogenase complex iron sulfur subunit B (SDHB) mutation, consistent with familial PG syndromes. Rapid progression to cardiogenic failure in a healthy child favors myocarditis, given her fever and sepsis. This is common in those who present with PC symptoms and elevated cardiac markers. In our review, most patients presenting in PC are older than 20 years, with cardiovascular emergencies [7]. They are often misdiagnosed with acute myocardial infarction (AMI). Authors suggested using coronary angiography for cardiac failure requiring ECMO to rule out AMI [7]. This patient had sudden onset symptoms of heart failure and cardiac arrest, with no evidence of a typical presentation. 

Our patient presented with elevated cardiac markers and echocardiography showing ventricular dysfunction, but PG was not diagnosed for almost two weeks, given that fulminant myocarditis explained her symptomatology. We focused on early recognition of cardiogenic shock, detailed investigations to rule out PC due to atypical presentations, and early anticipation of cardiopulmonary failure and ECMO as a lifesaving intervention.

PC involves catecholamine release acting on α-adrenergic receptors, causing arterial vasoconstriction and reduction in intravascular volume, with cellular ischemia leading to systemic effects. PC causes cardiovascular emergencies: myocardial infarction and catecholamine cardiomyopathy, progressing to circulatory collapse and shock. Myocardial injury includes damage to cardiac myocytes from increased myocardial oxygen demand and decreased oxygen supply, related to coronary artery spasms due to catecholamine release. PC treatment depends on recognition and stabilization until the cause is corrected, but medical treatment may not be sufficient. Cardiac emergency mechanical support, such as ECMO, has effectively treated numerous PC cases [8]. However, the ultimate treatment is surgical resection of the tumor. 

This case is essential, as it is the first report of a critically ill child warranting ECMO with no prior history or symptoms of PH, except on presentation. Our patient required resuscitation and emergent VA ECMO. The latter, although uncommon in acute myocardial dysfunction induced by catecholamine-induced shock in pediatric patients, has been reported in similar conditions, like takotsubo cardiomyopathy.

ECMO patients are at risk of fibrin-stranding and clot formation, contributing to embolic events. Catecholamine secretion contributes to clot formation via stimulation of platelet aggregation [5]. Myocardial dysfunction also contributes to stasis and clotting [5]. One complication experienced by our patient was multiple femoral arterial clots, necessitating above-the-knee amputation. Doppler showed reperfusion after repositioning; however, the above-mentioned factors and potential reperfusion injury likely contributed to clot formation after reperfusion. Our case review showed several cases of severe leg compartment syndrome in acute endocrinology crises requiring ECMO, despite the placement of a distal perfusion catheter. Most of these patients were older and had more prolonged catecholamine excretion than ours [5]. Placement of a larger diameter catheter, close monitoring of the catheter site, and repositioning of the lower artery catheter to the central common carotid artery were attempted without success in preventing the complications.

Preoperative management of PG is vital for preventing future complications. This is achieved with alpha- and beta-adrenergic blockade. The alpha blockade, over 16 days, and beta blockade, over 14 days, were initiated pre-operatively in our patient. Despite hypertension and tachycardia management, control of blood pressure <125/85 and heart rate <100 was not achieved before surgical removal of the tumor. She underwent an exploratory laparotomy and bilateral adrenal mass resection. The pathology report indicated a neuroendocrine tumor, favoring PH (pT3) in the left retroperitoneal region, with lymphovascular invasion and tumor presence at the margins. Additionally, a PG (pT3) was identified in the porta hepatis, also exhibiting lymphovascular invasion and tumor presence at the margins.

## Conclusions

Our patient presented with no history or provoking factors, despite an SDHB familial mutation identified after stabilization. VA ECMO can be a life-saving measure in such cases until a diagnosis is made. There is an increased risk of embolic events while on ECMO, and underlying catecholamine secretion further exacerbates clot formation. Continued reevaluation of the patient’s cardio-renal status led to the diagnosis without prolonged cardiac complications, but raised the importance of having a high index of suspicion for alternative causes of cardiac failure in the intensive care setting.
